# Education and Quality of Life: Does the Internet Matter in China?

**DOI:** 10.3389/fpubh.2022.860297

**Published:** 2022-03-18

**Authors:** Zhan Zhan, Zhi-Wei Su, Hsu-Ling Chang

**Affiliations:** ^1^Central China Normal University, Wuhan, China; ^2^School of Business, Wuchang University of Technology, Wuhan, China; ^3^Department of Accounting, Ling Tung University, Taichung City, Taiwan

**Keywords:** education, FDI, internet, quality of life, China

## Abstract

The internet has influenced human wellbeing through social networking, time-saving, diffusion of knowledge, and access to health information. Health is a key component of human quality of life. This study examines the nexus between education, the internet, and quality of life using data from China spanning the period from 1991 to 2020. The study used ARDL to examine the short and long-term, exploring education and the impact of the internet on quality of life. Education status plays a significant role in promoting quality of life in the short and long term. The empirical findings show the significant positive impact of the internet and ICT on quality of life in the short and long-run. Financial development and FDI improve the quality of life in the long-term in China. Based on these results, policymakers in China should develop the ICT infrastructure and human capital to support increased quality of life.

## Introduction

Human capital and economic productivity levels are closely related. Many researchers argue that the impact of education and awareness from using the internet positively impacts labor productivity. Human resources have robust effects on wages and the productivity of labor (1, 2). Moretti (3) noted that human resources have positive externalities by creating economic and social benefits. Berry and Glaeser (4) and Waldorf (5) also emphasize that the available level of human resources boosts quality of life. Shapiro (6) estimated that quality of life increases 40% of economic growth. Domestic human resources are important sources of economic development and it is vital to understand how areas differ in aggregate human capital stocks. In this case, one of the most crucial elements is the availability of college and university in those areas where the level of human capital is high (7).

In two ways, higher education institutes boost human capital stocks. They give easy access for people to higher education (8) and help attract people from other areas to get education in those institutions. They then stay in the areas where they received an education (9, 10). Winters (7) stated that the highly qualified population increase is due to student flow, especially when they are moving in search of high education and that growth has led to smart cities. People continue to live there because of a better quality of life and higher utilities.

Educational institutes attract many areas in many different ways. First, qualified people may be more likely to support native public goods like symphonies, parks, theaters, and museums. On the other hand, qualified immigrants could provide and run the diversity and density of consumer services such as coffee shops, bars, and restaurants, facilitating consumer requirements (5). A well-educated person could be more likely to use healthy foods and smart technologies for better health (11). A well-educated person mostly avoids crimes and participates in social activities (11), thus improving population health. The presence of higher education systems could therefore be said to be a critical amenity. The presence of these institutions helps to increase the level of educated people in society. This further enhances the quality of life of a society. Therefore, educational institutes boost the level of human resources that also affect the quality of life. Many other facilities and services are available in these areas, like book stores, bikes, records, bakeries, and restaurants (12). Hence the human resources and relative significance of higher educational institutions affect quality of life fundamentally.

The availability of ICTs has brought a tendance change in human lives (13). These have now become an essential tool in daily life. Consumers can do different jobs more speedily compared to the past. They can do business with e-commerce. With online banking systems, consumers and producers can manage financial matters (14, 15). They can chat easily with their friends and relatives and there are many more services they can easily access and use. Internet use I now widespread, but it also affects the behavior of users (16, 17). Several studies of the economics of happiness have applied wide-scale survey datasets to investigate the link between humans and ICTs (18), but many relevant potentially other channels through which use of internet designs the well beings have not been examined yet.

To date, research on this important issue lacks a clear and complete pattern that may join insights from other disciplines and facilitate future research.

The literature mostly focuses on social reality as ICTs transform people's lifestyles, especially in developing economies. ICT transforms the way we work, communicate, and operate in various segments such as education, health, livelihood, and other life activities (19). In short, internet diffusion and ICTs bring transformation in development and human behavior (20). In this modern era, the lives of individuals depend upon ICTS with remarkable economic and social impacts (21, 22). The outcomes of ICTs and internet diffusion may change at the regional level as the government regulation, infrastructure, and cultural changes influence the magnitude and type of the effects of ICTs (23). However, ICTs influence the daily lifestyle pattern of people at the individual level. ICTs and internet diffusion affect sustainable development at the macro level. Hence, directly and indirectly, ICTs and internet diffusion influence every aspect of life and contribute to improving quality of life (24, 25).

China is facing new types of health shocks that severely affect economic development. The population growth rate has been falling in recent decades (26). China's working-age population has a general decreasing trend in the economy. A number of researchers have noted that increasing quality of life is a new research agenda in China (27). It is therefore important for research to address the question “does education and digitization influence quality of life and if so, how and how much?” This study is the first to examine the nexus between the internet, education, and quality of life in China using data from 1991 to 2020. It aims to address gaps in previous empirical studies, which did not examine the short and long-run effects of the internet and education on quality of life (28). Previous literature was explored in a panel-wise analysis. The findings from this study are expected to be key to health practitioners and policymakers.

## Data

This study aims to examine the effect of education and internet diffusion on quality of life from 1991 to 2020. Table 1 delivers details about symbols, definitions of variables, and descriptive statistics. Data for all variables have been scrutinized by the World Bank, except HDI, education, and financial development. These variable data were obtained from UNDP, UNICEF, and IMF. Quality of life was measured through the human development index.

**Table 1:** Definitions and sources.

**Variables**	**Definitions**	**Mean**	**Median**	**Maximum**	**Minimum**	**Std. Dev**.	**Skewness**	**Kurtosis**
HDI	Human development index	0.653	0.654	0.795	0.510	0.087	−0.023	1.717
Education	Average years of schooling	11.30	11.25	15.21	7.394	2.389	−0.011	1.681
Hedu	School enrollment, tertiary (% gross)	22.15	19.65	58.42	2.806	17.68	0.663	2.142
Internet	Individuals using the Internet (% of population)	22.36	9.523	70.64	0.000	24.00	0.587	1.805
ICT	ICT index	26.32	19.56	73.56	0.152	25.09	0.457	1.712
FD	Financial development index	0.466	0.446	0.654	0.275	0.117	0.130	1.746
FDI	Foreign direct investment, net inflows (% of GDP)	3.389	3.500	6.187	1.139	1.341	0.068	2.382

The study used two proxies to measure education. The first is mean years of schooling, and the second is higher education (29). Internet diffusion is measured by the number of individuals using the internet as a percent of the population. ICT index is measured through internet users, mobile cellular, and fixed broadband. Besides these variables, the financial development index and foreign direct investment have been employed as control determinants. The mean of HDI, education, Hindu, internet, ICT, FD, and FDI is 0.653, 11.30 years, 22.15%, 22.36%, 26.32, 0.466, 3.389%, respectively.

## Model and Methods

The theoretical and empirical literature has proposed that education is vital for health outcomes. Education plays an important role in increasing the stock of human capital and likely has to improve quality of life. The internet also has favorable health outcomes through various mechanisms, such as health knowledge, self-awareness, e-health, and digital inclusion. Education and the internet have shared effects and separate impacts on quality of life (30, 31). The model is:


(1) $$\begin{array}{r} QOL_{2,t}=\;\delta_{0}+\;\delta_{1}Education_{t}+\delta_{2}Internet_{t}+\delta_{3}FD_{t}+\delta_{4}FDI_{t}+\varepsilon_{t} \end{array}$$

Where *QOL*_*t*_ is the quality of life that depends on educational attainment (Education), internet users (Internet), foreign direct investment (FDI), and financial development (FD). Education is an effective way to improve quality of life, thus sign of δ_1_ is likely to be positive. The internet enhances human health and wellbeing, thus ultimately positively affecting the quality of life; we expect an estimate of δ_2_ to be positive. Standard literature noted that quality of life is positively affected by financial development and FDI, thus δ_3_ and δ_4_ will be positive. Estimates of δ_1_, δ_2_, δ_3_, and δ_4_ reflect long-run effects of exogenous variables on the quality of life in the basic model, which does not incorporate short-term effects. We augmented model (1) in an error-correction format to assess long and short-run impacts in one step. The augmented model is:


$$\begin{array}{l} \Delta QOL_{t}=\;\delta_{0}+\;\sum_{k=1}^{n}\beta_{1k}\Delta QOL_{\;t-k}+\;\sum_{k=0}^{n}\beta_{2k}\Delta Education_{t-k}\\ \;+\sum_{k=1}^{n}\beta_{3k}\Delta Internet_{\;t-k}+\;\sum_{k=0}^{n}\beta_{4k}\Delta FD_{t-k}\\ \;+\sum_{k=1}^{n}\beta_{5k}\Delta FDI_{\;t-k}+\;\delta_{1}QOL_{t-1}+\;\delta_{2}Education_{t-1}\\ \;+\delta_{3}Internet_{t-1}+\;\delta_{4}FD_{2,t-1}+\delta_{5}FDI_{t-1}\\ \;+\lambda .\;ECM_{t-1}+\varepsilon_{t}eqnarray \end{array}$$

In Equation (2), β_1*k*_, β_2*k*_, β_3*k*_, β_4*k*_, and β_5*k*_ are short-term effects and δ_1_, δ_2_, δ_3_, δ_4_ *and δ*_5_ are long-run effects, δ_0_signifies the constant term, and ε_*t*_ represents error term. Nowadays, ARDL approach has commonly used in empirical research in time series analyses (32). The ARDL is a more flexible cointegration approach, which can be used in mixed integration I(0) and I(1). We used the DF-GLS test and Zivote Andrews unit root test with a structural break in the study. ARDL gives us short and long-run results in one step in different lag orders [Usman et al. (15) and Li et al. (33)]. This method does not enforce the condition that the macro variables have a similar order of integration. The confirmation of cointegration is based on the F-statistic and ECM. The null hypothesis of the *F*-test is, Ho: δ_1_ = 0, δ_2_ = 0, δ_3_ = 0, δ_4_ = 0, *and δ*_5_=0; while the alternative is H1: δ_1_≠0, δ_2_≠0, δ_3_≠0, δ_4_≠0;*and δ*_5_≠0. ARDL also gives us efficient results in small samples. We have applied the LM test for serial correlation. Breusch Pagan (BP) test for heteroskedasticity and the RESET test for model misspecification are also used to capture the econometric problems. Finally, we have applied the CUSUM and CUSUM-sq tests to confirm the stability of the parameter estimates.

## Results and Discussion

Before performing regression analysis, it is required to confirm the unit root properties of data. The study used DF-GLS and unit root tests with a break for this task. The coefficient estimates of both unit root tests are given in Table 2. DF-GLS test findings report that ICT and FDI are level stationary variables while the rest are I(1) stationary. However, unit root with break test findings reveals that FDI is a level stationary variable, and the other variables are first difference stationary. Based on the findings of both unit root tests, the study employed the ARDL approach to investigate the long-run association between dependent and independent variables. Table 3 displays the findings of all four ARDL models.

**Table 2:** DF-GLS and unit root with a break.

	**DF-GLS**			**Unit root with a break**				
	**I(0)**	**I(1)**	**Decision**	**I(0)**	**Break date**	**I(1)**	**Break date**	**Decision**
HDI	0.235	−4.253^***^	I(1)	−1.625	2018	−4.878^**^	2007	I(1)
EDUCATION	0.458	−4.587^***^	I(1)	−1.875	2003	−5.879^***^	1996	I(1)
HEDU	0.578	−2.655^***^	I(1)	−0.714	2012	−5.548^***^	2014	I(1)
INTERNET	0.215	−2.255^**^	I(1)	−1.452	2018	−4.987^***^	2006	I(1)
ICT	−1.923^*^		I(0)	−2.258	2016	−4.356^*^	2003	I(1)
FD	0.452	−5.235^***^	I(1)	−2.022	2001	−5.355^***^	2001	I(1)
FDI	−1.763^*^		I(0)	−4.256^*^	2011			I(0)

*** *p < 0.01*.

** *p < 0.05*.

* *p < 0.1*.

**Table 3:** ARDL estimates of quality of life.

	**Coefficient**	***t*** **-Stat**	**Coefficient**	***t*** **-Stat**	**Coefficient**	***t*** **-Stat**	**Coefficient**	***t*** **-Stat**
**Short-run**								
Education	0.019^***^	5.286	0.031^***^	5.268				
Education (−1)	0.005	1.365	0.010	1.418				
Education (−2)			0.002	0.459				
Hedu					0.001	1.369	0.001	1.272
Hedu (−1)					0.001^*^	1.821	0.003^*^	1.778
Hedu (−2)					0.001^*^	1.935	0.001^**^	2.201
Internet	0.000	0.642			0.002^***^	2.853		
Internet (−1)	0.002^*^	1.842			0.004^***^	3.554		
Internet (−2)	0.002^**^	2.062			0.002^***^	2.919		
ICT			0.002^**^	2.550			0.003^**^	2.051
ICT (−1)			0.001	0.548				
FD	0.027	1.151	0.062^*^	1.748	0.015	0.521	0.004	0.108
FD (−1)	0.045	1.707	0.072	1.533				
FD (−2)	0.024	0.992	0.032	1.048				
FDI	0.001	1.145	0.001	0.480	0.002	1.656	0.003^**^	2.136
FDI (−1)	0.002^*^	1.801	0.005	2.882				
**Long-run**								
Education	0.040^***^	11.84	0.062^***^	11.16				
HEDU					0.028^*^	1.738	0.027^*^	1.818
Internet	0.011^***^	3.285			0.013^*^	1.810		
ICT			0.021^***^	3.360			0.017^*^	1.834
FD	0.216	0.222	0.365^***^	4.379	0.079	0.145	0.050	0.105
FDI	0.015^***^	4.940	0.011^***^	7.992	0.013	0.655	0.034^*^	1.896
C	0.292^***^	9.470	0.204^***^	7.748	0.638^***^	2.988	0.631^***^	3.281
**Diagnostics**								
*F*-test	16.58^***^		15.42^***^		17.12^***^		11.25^***^	
ECM (−1)^*^	−0.364^***^	11.80	−0.482^***^	11.97	−0.378^***^	11.28	−0.393^***^	12.66
LM	0.875		1.745		1.542		1.587	
BP	1.254		0.874		1.302		1.925	
RESET	0.357		0.011		1.425		0.225	
CUSUM	S		S		S		S	
CUSUM-sq	S		S		S		S	

*** *p < 0.01*.

** *p < 0.05*.

* *p < 0.1*.

The long-run coefficient estimates of model 1 display that the impact of education and the internet is significant and positive on the quality of life, revealing that increase in educational attainment and internet diffusion lead to improvement in the wellbeing of society. The results report that in response to a 1 percent upsurge in educational attainment and internet diffusion, quality of life improves by 0.040 percent and 0.011 percent, respectively. Similarly, in the short-run, education reports a significant and positive impact on quality of life, but the internet has no impact on quality of life. The long-run coefficient estimates of model 2 infer that the increase in educational attainment and ICT results in improving the quality of life in China. It is found that a 1 percent escalation in educational attainment and ICT tends to enhance the quality of life by 0.062 percent and 0.021 percent. It shows that in the long-run, education and social networking are beneficial for human wellbeing in China. In contrast, the impact of education and ICT is statistically insignificant. This finding is also consistent with Alhassan and Adam (31), who noted that the internet had changed every aspect of human life. This means that the internet enhances human wellbeing by improving the quality of work-life, family life, leisure life, and community life.

These results are also in accordance with the structuration theory of DeSanctis and Poole (34), which noted that digital inclusion positively influences human wellbeing by improving social life. This infers that the presence of digitalization allows individuals to enhance their happiness and increase social networking, thereby improving their wellbeing. Digitization diffusion can also reduce corruption in government by providing efficient services for the people and thereby increasing their human wellbeing. This finding also infers that internet use promotes human wellbeing by enhancing psychological and physical wellbeing, facilitating social networking, and encouraging self-esteem. These transmission channels are also supported by other studies (35, 36). The internet can also improve human wellbeing via income chandelles, as supported by another study (37).

In model 3, the long-run coefficient estimates infer that tertiary education and the internet are positively and significantly associated with quality of life, displaying that improvement in quality of life occurs due to an increase in tertiary education and internet diffusion. A 1 percent increase in the level of tertiary education and the internet results in an improvement in quality of life by 0.028 percent and 0.013 percent. The impact of tertiary education was found to be insignificant in the short-run. However, internet diffusion brings an improvement in quality of life in the short-run. In model 4, findings revealed that tertiary education and ICT exert a significant and positive impact on quality of life in the long-run. It revealed that a 1 percent upsurge in tertiary education and ICT brings improvement in quality of life by 0.027 percent and 0.017 percent, respectively. Tertiary education reports no significant impact on quality of life in the short-run. However, ICT is positively and significantly associated with quality of life in the short-run.

Education estimates are also reliable with Michalos (30), who noted that education, directly and indirectly, influences human wellbeing. Education enhances happiness and quality of life. Education has only a small direct effect on happiness. Education reduces poverty and income inequality, which in turn improves human wellbeing. This means that education increases chances of life success; thus, it also impacts quality of life (38). Education directly influences occupational status in the economy, thus improving the overall quality of life. The theoretical literature also considers that education is a key pillar of human health and wellbeing. This finding is also supported by Li and Ullah (39), who noted that education has some benefits for physical and psychological health.

This study incorporated the role of financial development and foreign direct investment on quality of life. The findings display that financial development is positively associated with quality of life only in model 2 in the long and short-run. However, foreign direct investment is positively associated with quality of life in all models except model 3 in the long-run. These findings demonstrate that the inflow of foreign direct investment is beneficial for improving quality of life, as the FDI leads to generating new employment opportunities that increase the consumption and income level, thus contributing to enhancing the wellbeing of households. The impact of FDI is positive and significant on quality of life only in model 4 in the short-run. In the lower panel of Table 3, the empirical estimates of some important diagnostic tests are given. The *F*-test and ECM tests confirm the existence of a long-run cointegration association among variables in all four models. No consequences of autocorrelation and heteroskedasticity are detected in any model. The coefficient estimates of the Ramsey RESET test confirm the correct specification of models in all four regressions. The stability of all four models is confirmed through the findings of CUSUM and CUSUM-sq tests.

## Conclusion and Implications

Since the 1990s, rapid spreads in ICT have had a profound effect on many societies and countries. Development organizations and government agencies believe that ICT can play a significant role in improving quality of life. There is vast theoretical and empirical literature documenting the transmission channels and effects of ICT in economic development, but less attention has been given to the effects of ICT diffusion in human development in the digital era.

This study contributes to ICT diffusion theory and literature on human development. It examines the impact of education and the internet on quality of life using time series data from China gathered between 1991 and 2020. We employed the theoretical model given by DeSanctis and Poole (34), using social factors as inputs to quality of life. This study confirms that increased internet diffusion improves the quality of life in the short and long-run. This is because digital diffusion leads to good relationships and healthy behaviors among individuals, thereby improving their quality of life. ICT affects quality of life through increased social networking, improving leisure life, work-life, and family life, and increasing income levels of individuals. Similarly, education has a positive and significant impact on quality of life in the long-run. The findings are sensitive to a variables-based robust method. This means that education is a vital cog in the human development engine in China. The findings also validate the significant positive effect of financial development on quality of life and also reveal that FDI significantly increases the quality of life.

### Implications and Limitations

The findings of the study have some important implications. Policymakers should also design policies that confirm ICT diffusion in society because it also improves human development by improving health literacy. Policies that enhance human capital and facilitate digital inclusion can also create well health outcomes. To address digital diffusion, policymakers should also emphasize demand-side issues and supply-side aspects. Based on the findings, we propose that ICTs policies need to be designed so that the positive effects of education can be increased. Health care programs need to be aligned with economic policies that ensure higher education attainment. Governments should also invest more in ICT infrastructures and educating the rural public on the use of ICT applications could be a robust policy to improve quality of life. As China faces enormous new challenges in public health, the government should increase green fiscal spending on education, health, and technology sectors in China to address them.

This study could not analyze the impact of education and ICTs diffusions on the quality of life at the aggregate level. Future research should use different measures of ICTs diffusions such as mobile cellular and fixed broadband. The present study is only limited to the quality of life but ignores happiness as dependent on empirical analysis. Future research may also extend this analysis to China to measure the determinants of happiness. The study can also be extended empirical analysis for China with primary data.

## Data Availability Statement

Publicly available datasets were analyzed in this study. This data can be found here: https://data.worldbank.org/.

## Author Contributions

ZZ: conceptualization, software, data curation, and writing-original draft preparation. Z-WS: methodology, writing-reviewing, and editing. H-LC: visualization and investigation. All authors contributed to the article and approved the submitted version.

## Conflict of Interest

The authors declare that the research was conducted in the absence of any commercial or financial relationships that could be construed as a potential conflict of interest.

## Publisher's Note

All claims expressed in this article are solely those of the authors and do not necessarily represent those of their affiliated organizations, or those of the publisher, the editors and the reviewers. Any product that may be evaluated in this article, or claim that may be made by its manufacturer, is not guaranteed or endorsed by the publisher.
